# External quality assurance program for diagnostic complement laboratories: evaluation of the results of the past seven years

**DOI:** 10.3389/fimmu.2024.1368399

**Published:** 2024-03-26

**Authors:** Michael Kirschfink, Ashley Frazer-Abel, Emese Balogh, Sabine Goseberg, Nathalie Weiss, Zoltán Prohászka

**Affiliations:** ^1^ Institute of Immunology, University of Heidelberg, Heidelberg, Germany; ^2^ Instand eV, Düsseldorf, Germany; ^3^ ExseraBioLabs, University of Colorado, Aurora, CO, United States; ^4^ Department of Pharmaceutics, Semmelweis University, Budapest, Hungary; ^5^ Department of Internal Medicine and Hematology, Füst György Complement Diagnostic Laboratory, Semmelweis University, Budapest, Hungary

**Keywords:** external quality assessment schemes, proficiency testing, complement pathways, functional analysis, complement activation products, autoantibody, C3 nephritic factor

## Abstract

**Introduction:**

The complement external quality assurance (EQA) program was first organized in 2010 by a group of researchers working in diagnostic complement laboratories. Starting in 2016, INSTAND e.V., a German, non-profit interdisciplinary scientific medical society dedicated to providing expert EQA programs for medical laboratories, started organizing the EQAs for complement diagnostic laboratories together with the same group of experienced scientists and doctors who also work as EQA experts. The aim of the current work is to provide descriptive analysis of the past seven years’ complement EQA results and evaluate timeline changes in proficiency testing.

**Methods:**

Each year, in March and October, blinded samples (normal, pathological) were sent to the participating diagnostic laboratories, where complement parameters were evaluated exactly as in daily routine samples. Since no reference method/target values exist for these parameters, and participants used different units for measurement, the reported results were compared to the stable mean (Algorithm A) of the participants using the same method/measurement units. A reported result was qualified as “passed” if it fell into the 30-50% evaluation/target range around the mean of reported results (depending on the given parameter).

**Results:**

While the number of participating laboratories has increased in the past years (from around 120 to 347), the number of complement laboratories providing multiple determinations remained mostly unchanged (around 30 worldwide). C3, C4, C1-inhibitor antigen and activity determinations provided the best proficiency results, with >90% passing quotas in the past years, independent of the applied method. Determination of the functional activity of the three activation pathways was good in general, but results showed large variance, especially with the pathological samples. Complement factor C1q and regulators FH and FI are determined by only a few laboratories, with variable outcomes (in general in the 85-90% pass range). Activation products sC5b-9 and Bb were determined in 30 and 10 laboratories, respectively, with typical passing quotas in the 70-90% range, without a clear tendency over the past years.

**Conclusion:**

With these accumulated data from the past seven years, it is now possible to assess sample-, method-, and evaluation related aspects to further improve proficiency testing and protocolize diagnostic complement determinations.

## Introduction

As a major part of innate immune system, complement is not only essential to fight invading pathogens but also plays a key role in immune surveillance, homeostasis and repair (1–3). More than 50 soluble and cell-bound proteins serve either as danger sensing molecules for invading pathogens, apoptotic and necrotic cells and immune complexes (e.g. C1q, mannose-binding lectin/MBL, ficolins, properdin, proteins of the Factor H family). These molecules act within an enzymatic cascade and provide a very effective regulation of immunity via receptors on the surface of multiple immune-, and tissue cells (for review see (4, 5). Complement proteins in the circulation are primarily synthesized in the liver and by monocytes, but are also constitutively expressed and secreted by many other cell types in different tissues into the microenvironment (6, 7).

**Figure 1:**
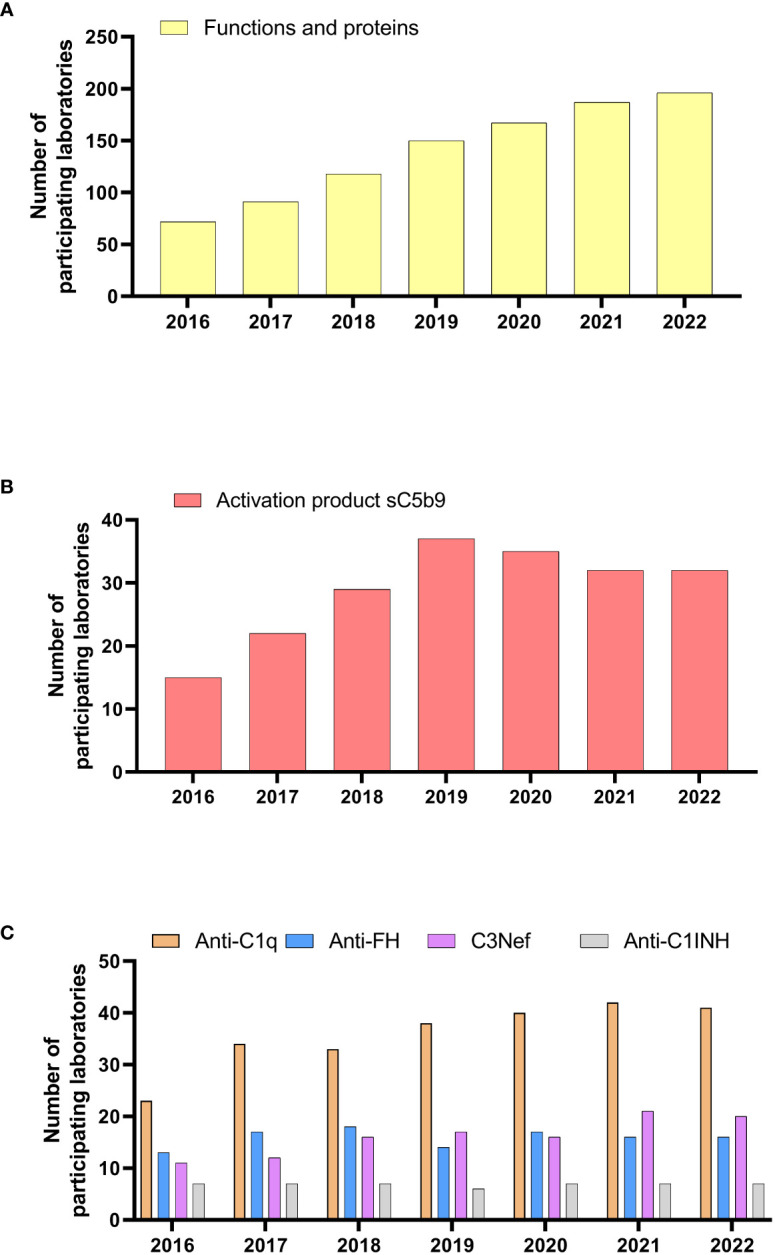
Numbers of participating laboratories in the different complement EQA programs in the past years. **(A)** EQA246, **(B)** EQA247, **(C)** EQA245, EQA248,EQA249 and EQA250. For EQA245 and EQA246 numbers of laboratories with at least 1 participation in the indicated year are given.

Upon activation via the classical (CP), lectin (LP) or alternative pathway (AP), the central components C3 and C5 are cleaved, which results in the opsonization of pathogens and debris with C3b and iC3b, the recruitment of inflammatory cells via the anaphylatoxins C3a and C5a, and finally in the formation of the membrane attack complex, C5b-9 (reviewed in (8–10). Under physiological conditions, the system is tightly regulated by proteins in the fluid phase (CP: C1-inhibitor, C4 binding protein, Factor I; AP: Factor H, Factor I; LP: C1-inhibitor, terminal pathway: clusterin, vitronectin/S-protein). Membrane-bound inhibitors protect each individual cell in the circulation and solid tissue (CD35/CR1, CD46/MCP CD55/DAF and CD59) to prevent unwanted activation (11, 12). For schematic illustration of complement pathways and regulation see **Figure 1** in (13).

While the primary action of complement is well described for plasma and body fluids in the extravascular space, more recent studies suggest a possible role also inside the cell (14). Multiple interactions exist between the coagulation, fibrinolytic and complement systems where enzymes can cleave and activate one another, and regulators are shared between cascades (15). This provides a good explanation why many complement-driven diseases (e.g. PNH, aHUS, CHAPLE syndrome) express thrombosis as a hallmark of clinical manifestation (16, 17).

Complement deficiencies comprise about 5-10%, according to different registries of all primary immunodeficiencies with a combined genetic prevalence of 0.03% in the general population. Probably clinically more relevant are consequences of complement overactivation leading to numerous inflammatory and autoimmune diseases (18–20).

In the last decades great progress has been made in complement analysis to not only understand its physiology but also to better define disease development, severity, and response to therapy (21). This has been further accelerated by the introduction of complement-targeting drugs, which has led to a significant increase of interest by clinicians (13, 22).

A comprehensive laboratory analysis of the complement system should start with the assessment of the total activity of the classical and alternative pathway either by functional ELISA or by hemolytic or liposome-based assays (23). These global tests provide information about the integrity of the entire complement cascade. A missing or greatly reduced activity indicates a primary complement deficiency but may also be due to a secondary deficiency caused by decreased synthesis, increased consumption, or protein loss. Deficient or dysfunctional proteins of the affected pathway are identified by single protein (ELISA, radial immunodiffusion, immunoelectrophoretic or nephelometry/turbidimetry) or functional tests (24, 25).

Since most of the complement components are acute-phase proteins with a higher rate of synthesis in inflammation, in acute-phase reaction individual components are often left within the normal range despite ongoing consumption. Only the analysis of complement activation products allows one to distinguish with enough sensitivity complement deficiency from pathologically increased complement activation and consumption *in vivo (* 26). Complement activation products may be either split fragments after enzymatic cleavage of certain components, e.g., C4 (C4a, C4b/c, C4d), C3 (C3a, C3b, iC3b, C3c,C3dg, C3d), FB (Ba, Bb), and C5 (C5a), or protein complexes where activated components are bound to their respective regulators, like C1rs–C1-INH, the properdin-containing alternative pathway convertase C3bBbP, and sC5b-9 (soluble terminal complement complex, also known as soluble membrane attack complex sMAC, or terminal complement complex TCC). Quantification can be done as traditional ELISA, upon binding to high-capacity immunosorbent with subsequent elution, or to microbeads applied in multiplex flow cytometric technology (see below). Those neoepitope-specific antibodies are also valuable to detect *in situ* complement activation applying immunohistochemistry.

Sensitive and quantitative multiplex analysis tests are currently developed to simultaneously assess multiple complement proteins and activation products but have not yet been applied to routine complement analysis (27).

Importantly, routine laboratory analysis of complement abnormalities also involves the measurement of clinically relevant inhibitory or activating autoantibodies targeting individual complement components, regulators, or convertases such as C1 inhibitor, C1q, Factor H, and C3 nephritic factor. These autoantibodies have been demonstrated to be useful as diagnostic or prognostic markers as well as for monitoring therapeutic responses (28).

Preanalytical considerations are important determinants of quality of results in the diagnostic complement laboratory (29). As outlined in a recent review by Brandwijk et al. about 50% of all investigated studies failed to use the right sample type or technique (30).

Since many complement proteins are heat labile precise preanalytical sample handling is mandatory for accurate and conclusive laboratory complement diagnostics. Correct collection and processing of all body fluid samples for complement analysis is essential to avoid artificial *ex vivo* complement activation. Without inhibition, physiological and pathological complement activation continues *ex vivo* obscuring the actual complement activation status and preventing meaningful data interpretation.

Serum is the appropriate sample to measure complement activity, components, regulators, and autoantibodies. It should be separated by centrifugation after full clotting and samples should be used immediately or can be stored at -70 °C for longer times. Since multiple serine proteases from other cascade systems can cleave complement components it is strongly recommended to use EDTA-plasma for analyzing complement activation products. Heparin and citrate-based anticoagulants are less useful (31).

For most complement activation products, EDTA-plasma is stable for up to 4 hours at room temperature (32) but should better be kept on ice or in a refrigerator if analyzed on the same day. For later processing, the sample should be aliquoted, frozen, and stored at -70 °C. Frozen samples should be thawed at room temperature or on ice, but not in a water bath at 37 °C. Repeated freezing and thawing of aliquots should be avoided. Frozen samples must be shipped on dry ice by courier if transport is necessary. Samples must be collected prior to plasma infusion or plasma exchange, or before any kind of immune therapies causing complement mediated cytolysis (for example anti-CD20 or anti-thymocyte globulin therapies) to determine the initial disease-related complement status.

In urine, the measurement of complement activation products can be affected by high amounts of urea and urine proteases. Since activation products in proteinuria may appear as a consequence of extrarenal (artificial) rather than intrarenal complement activation, the addition of protease inhibitors is required (33).

Complement proteins can also be analyzed in bronchoalveolar lavage (34), cerebrospinal (35) or synovial fluids (36) as well as tears and aqueous/vitreous humor (37) which may better reflect a local complement activation.

Finally, correct interpretation requires validated reference intervals. Here it should be emphasized that the reference intervals for several components are age-related, especially when analyzing samples from infants this must be taken into account (38–40).

Following the increased attention for complement analysis over the last 2 decades and a need to improve its consistency and quality the Sub-Committee for the Standardization and Quality Assessment of Complement Measurements was established and formally recognized by the IUIS (https://iuis.org/committees/qas/subcommittee-for-the-standardization-and-quality-assessment-of-complement-measurements/). Since 2010, 20 rounds of external quality assessment (EQA), now covering up to 20 parameters (function, proteins, activation products and autoantibodies) have been completed. The aim of the current work is to provide descriptive analysis of the past seven years’ complement EQA results and evaluate timeline changes in proficiency testing.

## Methods

### Sample materials – properties and preparation

In each EQA survey, two samples with normal or pathological concentrations/activities of complement parameters were distributed to the participating laboratories for quantitative or qualitative analysis (**Supplementary Table 1**). The samples were obtained from either voluntary blood donors or from patients. The samples tested negative for HIV, HBV, and HCV. No stabilizing additives were added (except for EQA247 where EDTA-anticoagulated plasma sample is provided). Samples were lyophilized due to stability reasons for EQA schemes EQA246 and EQA247, and since 2022 also for EQA248. Before 2018 the samples were lyophilized by a commercial provider (in.vent Diagnostica GmbH, Henningsdorf, Germany). Afterwards, the process was done in the Department of Pharmaceutics at Semmelweis University: 1.0 mL in case of EQA246, otherwise 0.3 mL were aliquoted in polypropylene cryo tubes (1.0 and 0.5 mL; Sarstedt, Nümbrecht, Germany). Before 2022, sample volumes differed based on the survey (**Supplementary Table 1**). The freeze-drying was performed in a one-chamber type equipment (ScanvacCoolsafe 110-04, LaboGene™, Lynge, Denmark) containing a two shelf sample holder. The process was controlled by a computer program, the temperature and pressure values were recorded continuously. The temperature of the drying chamber was between -97 °C and -95 °C for successful condensation. The samples were previously frozen and kept at -70 °C until the start of lyophilization. The lyophilization started at -40 °C for 1 hour, then temperature of the shelf was increased to the range between 0 °C and 30 °C for 18 hours during the primary drying under 0.02-0.03 hPa vacuum. The secondary drying was performed at 40 °C shelf temperature for 3 hours, where the sample temperature did not exceed 10 °C. The entire lyophilization process took 22 hours. The stability and homogeneity of EQA samples were confirmed according to DIN EN ISO/IEC 17043:2023. All samples were stored at -18 °C until dispatched to participants at ambient temperature.

### Ethics statement

The patient’s informed written consent is available for the project. A positive vote from the Scientific and Research Committee of the Medical Research Council of Hungary has been obtained. The study was conducted according to the declaration of Helsinki.

### EQA procedure

The INSTAND e.V. EQA schemes for analyzing complement parameters are offered worldwide once or twice per year, depending on the scheme. EQA schemes, that are only provided once per year are shipped in October (O) and EQA schemes, that are provided twice a year, are shipped in March (M) and October (O). For detailed information on the different parameters included in each EQA scheme, see **Supplementary Table 1**. Participating laboratories provide their laboratory results and information on the respective method and reagent provider via the platform RV-Online (http://rv-online.instandev.de). For the evaluation of quantitative results, the consensus value (stable mean) of all participants, calculated using algorithm A, was used (41). Evaluation area around this consensus value depended on the parameter. Detailed information can be found in **Supplementary Table 1**. With respect to the qualitative results, the participants had to indicate whether the samples were positive, borderline, or negative. The evaluation of qualitative results is based on prior expert evaluation in the laboratory providing the test material.

Qualitative EQA data can be found in **Supplementary Table 2** and quantitative EQA data in **Supplementary Table 3**.

When a manufacturer-dependent variance was observed, collectives were formed and evaluated separately.

### Data analysis and statistics

Data are presented as numbers (%) of participants and mean (with SD) of passing quotas (for samples, or for groups). Sample performance rates (passing quota,%) were calculated in the following way: number of laboratories providing results in the target range for a given sample, divided by the number of all laboratories providing results for that given sample. Total rate (passing quota of the group) was calculated in the following way: number of laboratories providing results in the target range for both samples, divided by the number of all laboratories presenting results for both samples.

Statistica 13.5 and GraphPad Prism 9 softwares were used for statistical analysis and data presentation.

## Results

### The complement EQA program, participation

**Figure 2:**
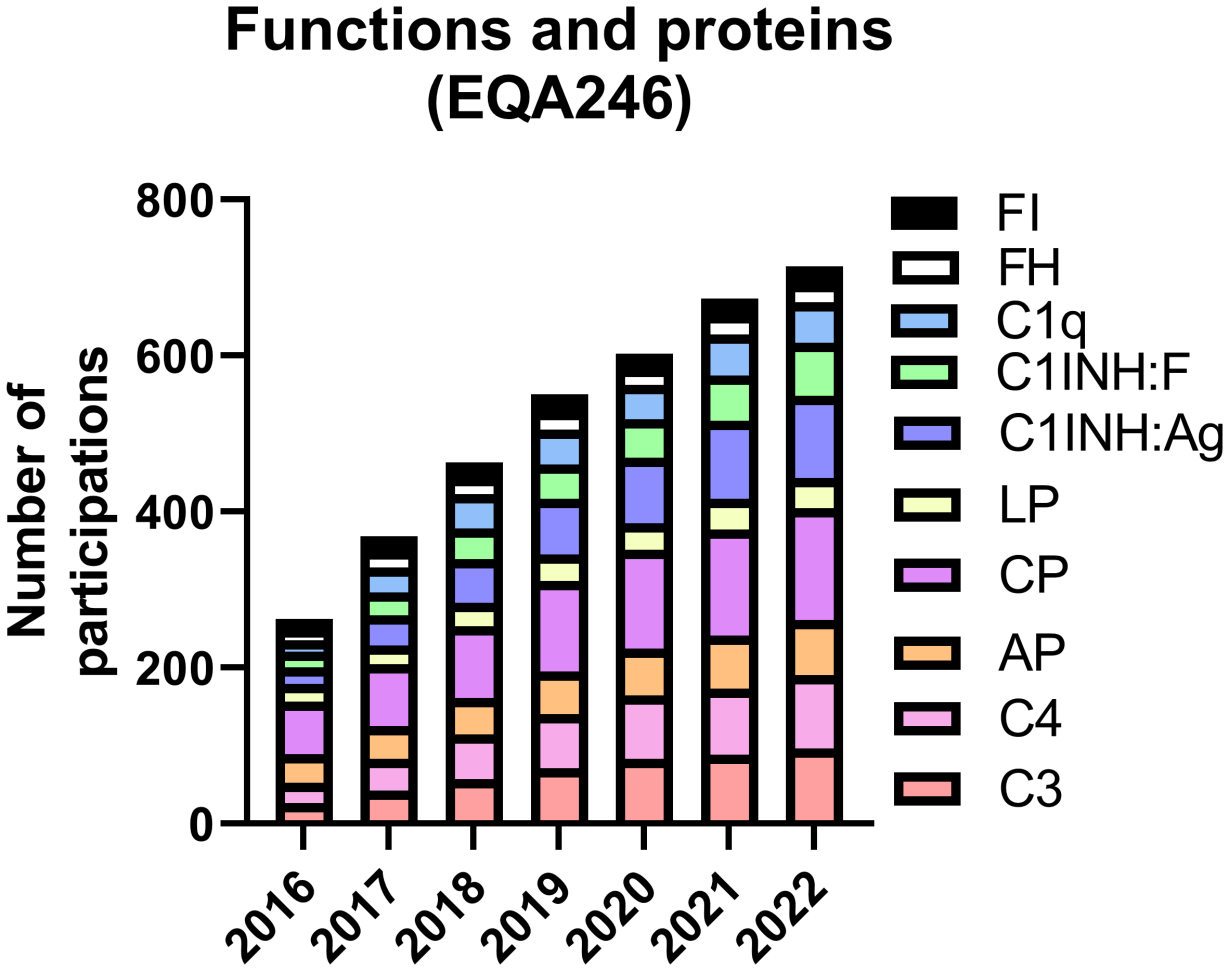
Cumulative number of participations (split by various parameters) from laboratories submitting at least one result in the indicated year. Note, that EQA246 was offered twice a year, and the majority of the participants submitted results twice.

The EQA program of diagnostic complement laboratories comprises six schemes: EQA246 (ten parameters) for complement function, components, and regulators, EQA247 (four parameters) for complement activation products, EQA245 for IgG anti-C1q autoantibody, EQA248 for C3-nephritic factor, EQA249 for IgG anti-FH autoantibody and EQA250 (three parameters) for anti-C1-inhibitor autoantibodies (see **Supplementary Table 1**). For EQA246, participation markedly increased by 170% in the past seven years (**Figure 1A**), with almost three times more laboratories participating in 2022, as compared to 2016. The highest increase in participation was observed for C3, C4, C1q, C1-inhibitor concentration (C1INH : Ag) and C1INH function (C1-INHF) (**Figure 2**). This contrasts with EQA247, where only the terminal pathway activation marker sC5b-9 was measured in at least eight laboratories per year (**Figure 1B**). Participation peaked in 2019 with a small decline afterwards. Participation for the complement related autoantibodies show great variance (**Figure 1C**). For anti-C1q and C3Nef there is a clear increase (by 78% and 81%, from 2016 to 2022, respectively). Participation for anti-FH and anti-C1INH remained unchanged in the past years.

For each of the six complement EQA schemes, two blinded samples were offered to the participants: one with normal/negative, and a second with pathological/positive parameter level. Since no reference method or target values exist for these parameters, and participants used different units for their data, the reported results were compared to the stable mean of the participants using the same method/measurement units, if there were at least eight participants in that given subgroup. A reported result was qualified as “passed”, if it fell within the 30-50% range around the stable mean (depending on the given parameter). For autoantibody determinations the participants had to report qualitative results using their own cut-off values. In the next paragraphs, EQA performance results are reported as passing quota, indicating the percentage of participants having “passed” in a given EQA scheme. Note, that passing quota was not calculated for subgroups with fewer than eight participants.

### Performance of the participants for complement function and proteins

**Figure 3:**
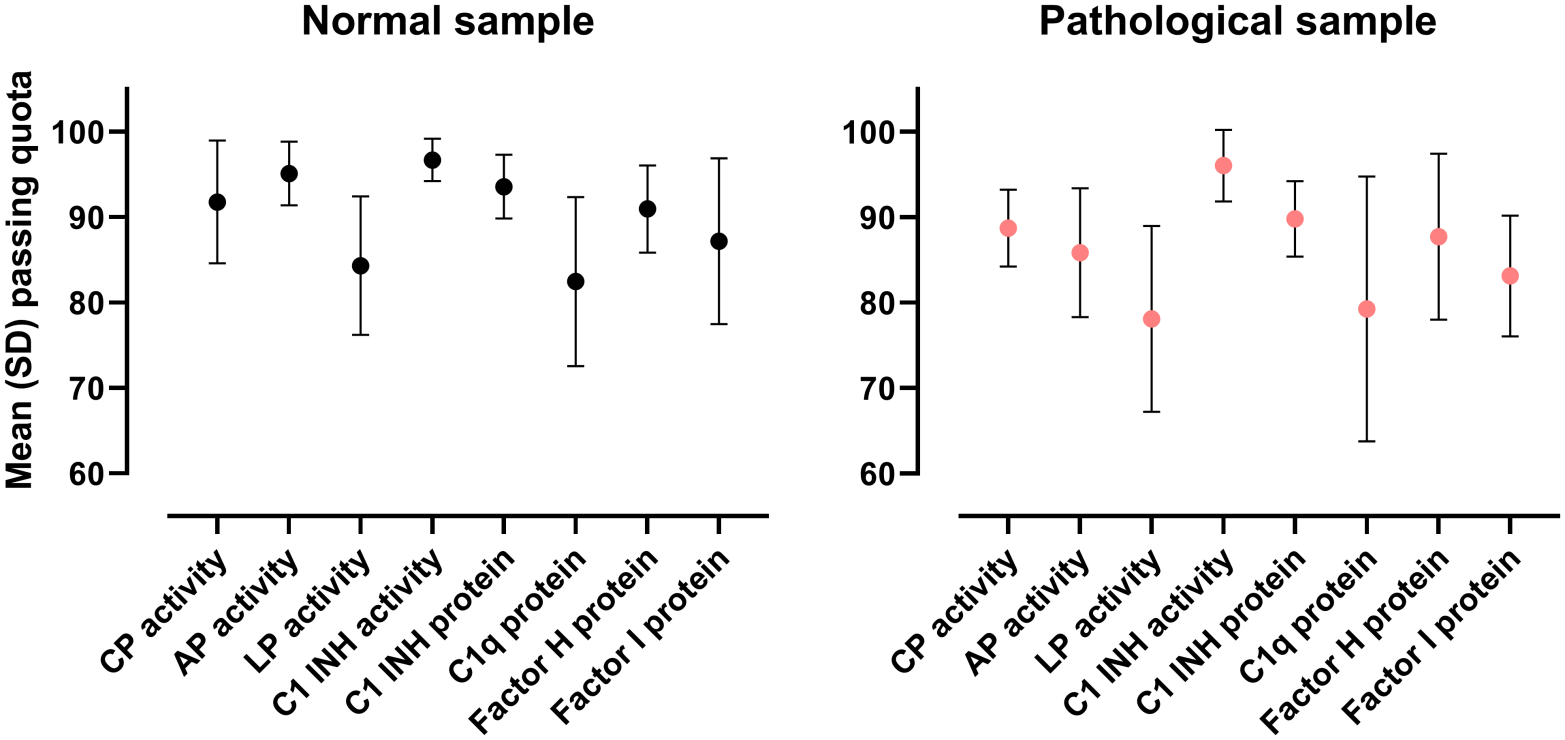
Overview of the complement function and proteins (EQA246) EQA results. Data shown are means with standard deviation of the results obtained in the 12 surveys between 2017 and 2022.

**Figure 4:**
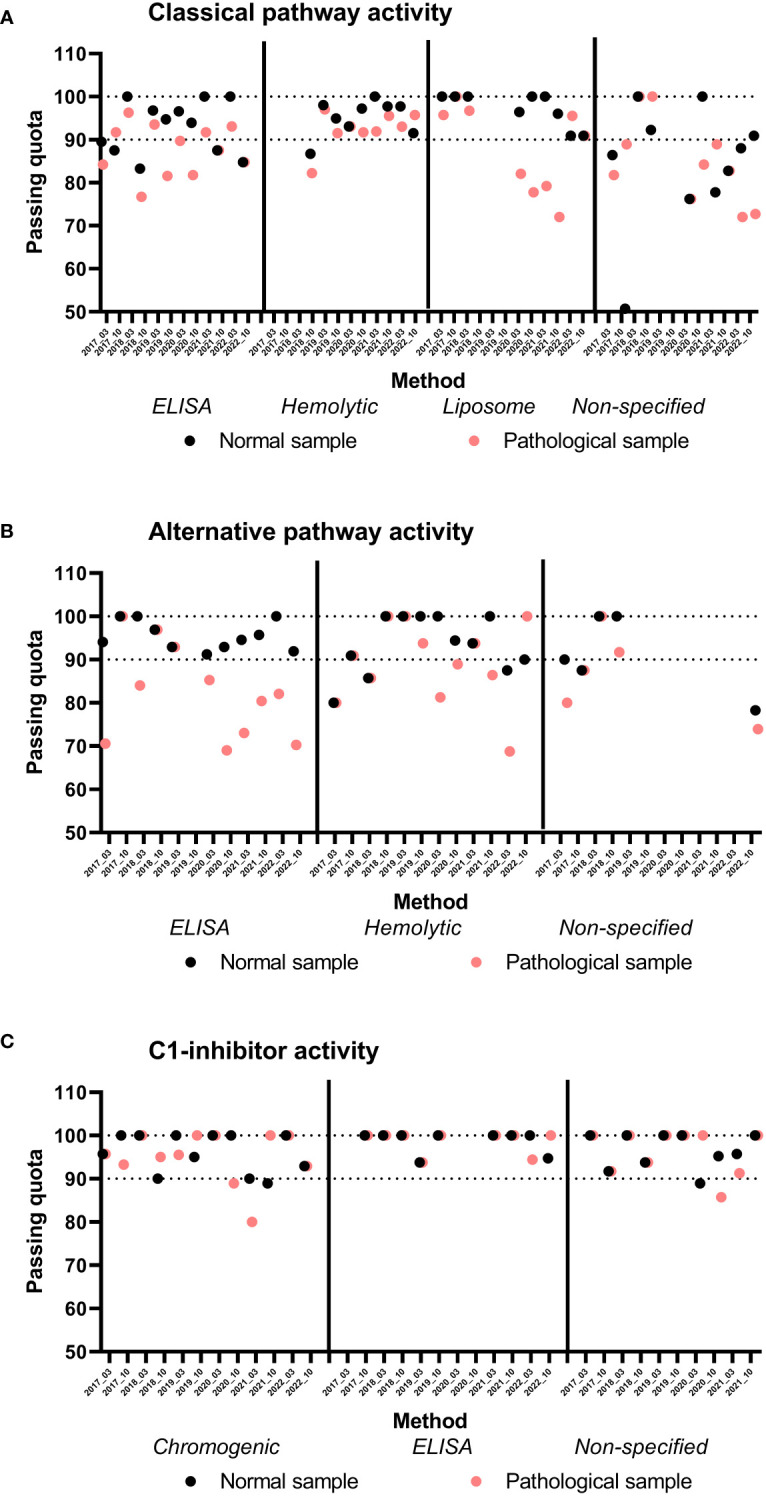
Results of classical **(A)**, alternative pathway functional activity **(B)**, and C1-inhibitor activity **(C)**, split by assay methods and EQA surveys. Passing quota of the indicated samples are plotted for indicated EQA surveys and assay groups. Note, that groups with less than 8 participants are not analyzed.

**Figure 3** shows mean (with SD) passing quota of 2017-2022 results for EQA246 parameters, separately for the normal (pool of healthy blood donor’s serum samples) and the pathological (mixture of normal and heated serum sample of healthy donors) samples. Best performing tests were those for C3 and C4 (not presented, passing quota all the time above 90%, mainly measured by nephelometry or turbidimetry). For C1-inhibitor antigen (measured mainly by nephelometry or turbidimetry) and -function a comparable good performance was observed. The passing quota only occasionally fell below 90%. For C1-inhibitor function approximately two-thirds of the participants used a chromogenic assay (manual or automated), whereas one third used a functional ELISA, both methods and all platforms providing consistently good outcomes (**Figure 4**).

For determination of complement activity (CP, AP and LP), passing quota on average was higher for normal than for the pathological samples (**Figure 3**), and this observation is almost constantly present across the years (**Figure 4**, for CP and AP). CP activity was measured about equally often by each of three methods, based on sheep red blood cell (SRBC) hemolysis, on liposome lysis, or on functional ELISA (detection of C9-neoepitope). **Figure 4** shows passing quota separately for these three methods for the last six years (12 EQA surveys). We observed a high variance (70-80% to 100%), with a slightly better performance for the hemolytic assay. Results for AP activity determination were similar in the range of 70% to 100%, without a clear trend or difference in the data over the years or method subgroups (hemolytic or functional ELISA based method).

**Figure 5:**
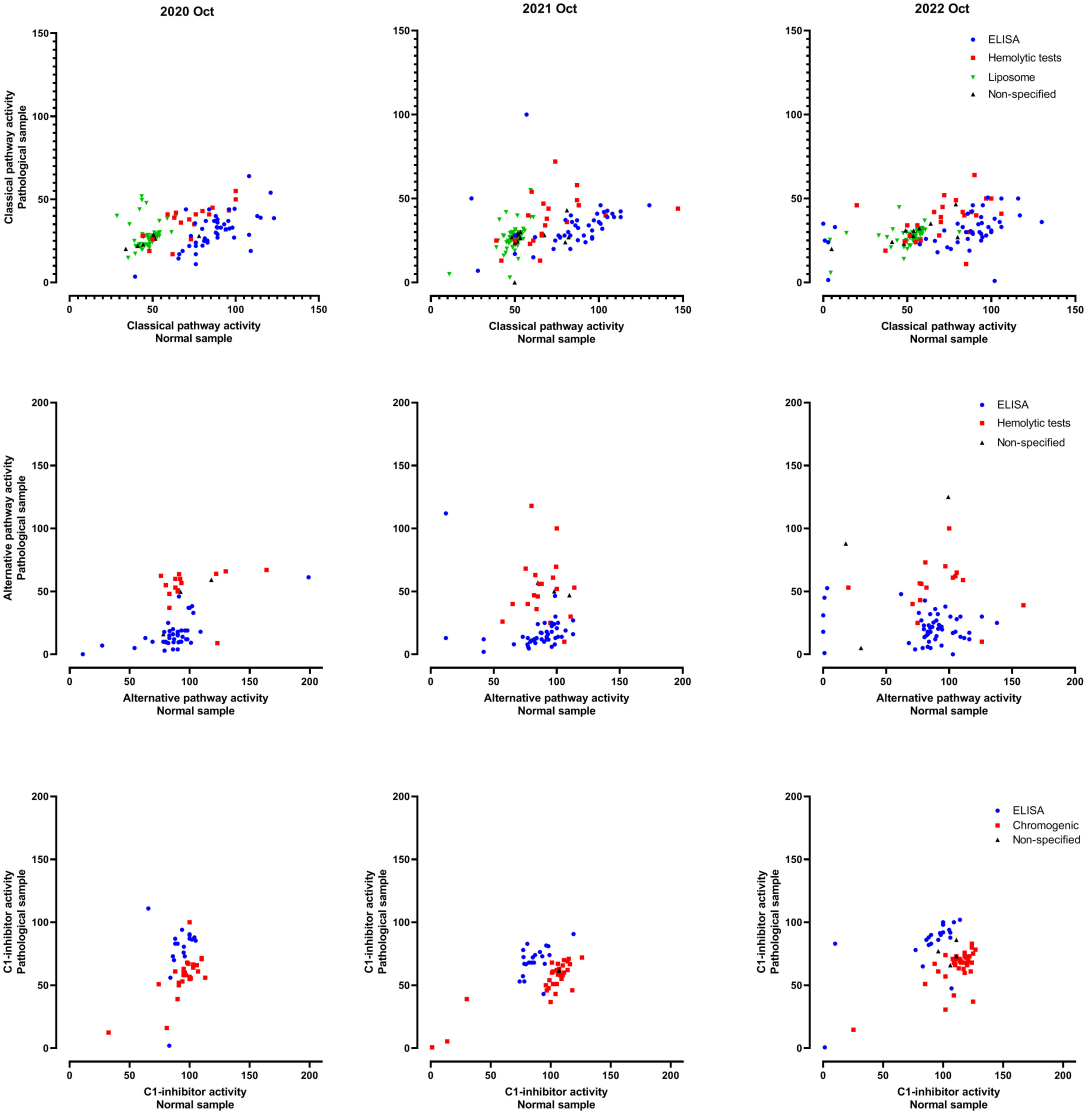
Individual results of classical and alternative pathway functional activity, and C1-inhibitor activity. Data shown are activity results (%) of the normal and the pathological sample, assay methods are indicated by the symbols/colors.

Several efforts were made in the past to harmonize functional testing in the complement laboratories, either by assay calibration (test sample compared to a normal pool assigned as 100%) or scaling (percentage). Furthermore, the various functional assays yielded nearly similar performance results for C1-inhibitor function, CP and AP activity (**Figures 3**, **4**). However, despite these efforts the raw data from the past years remain divergent between the different functional methods (**Figure 5**) which indicates that those measurements and results are not interchangeable.

### Performance of the participants for complement activation products

Four parameters (C3a, C3d, Bb and sC5b-9) are included in scheme EQA247 for complement activation product, in which two blinded, lyophilized samples (0.5 mL normal EDTA plasma, 0.5 mL EDTA plasma spiked with serum of the same donor) are sent to the participants.

**Figure 6:**
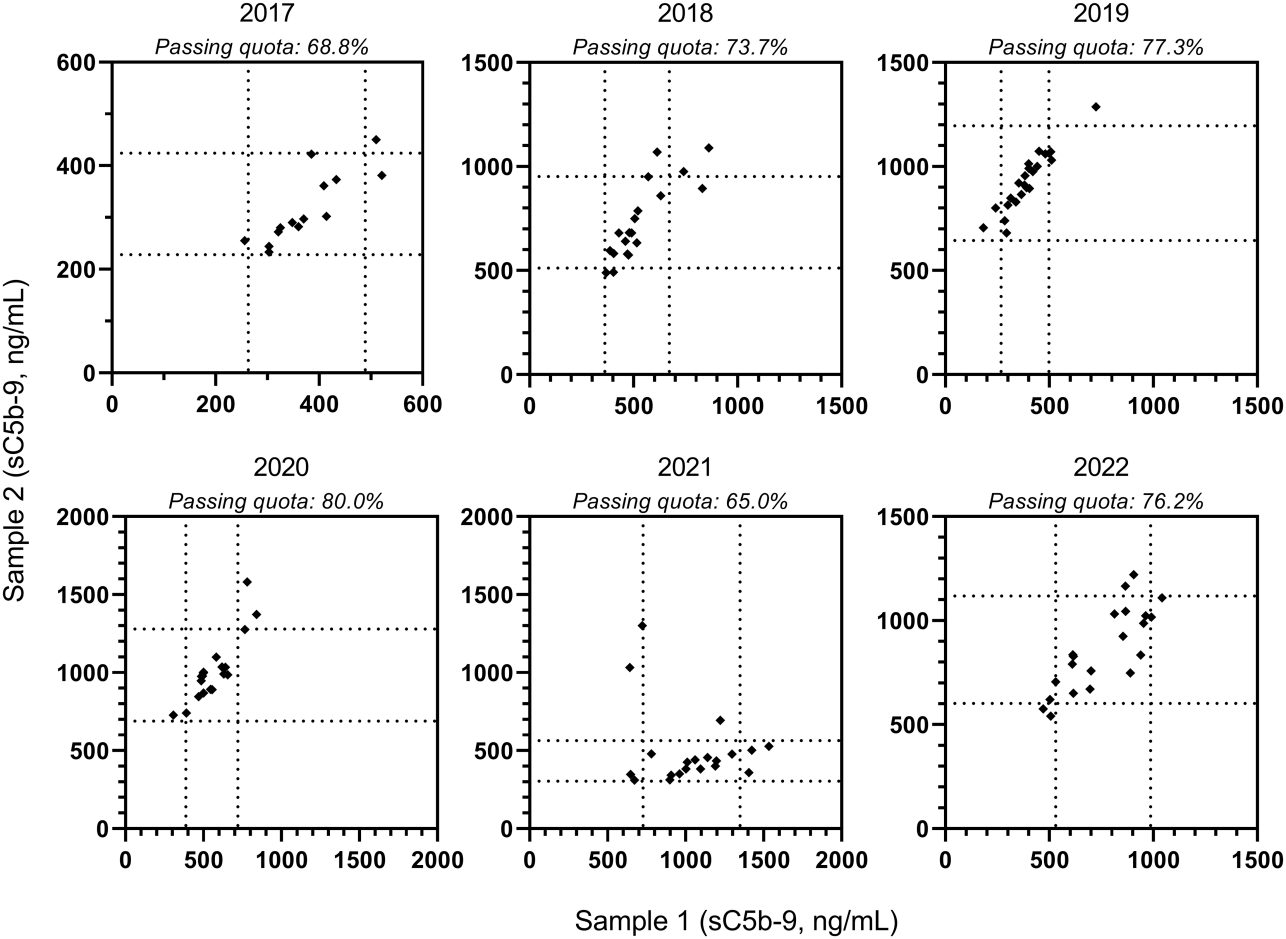
Individual results of terminal pathway activation marker (sC5b-9) levels, measured by ELISA assay of Quidel. Data shown are sC5b-9 results of the low and the high concentration samples, as obtained in the past six EQA surveys. Dotted lines indicate acceptance limits for the samples; passing quota (both of the results “passed”) of the collective is indicated above the figure for the different years.

**Figure 6** illustrates primary measurement results for sC5b-9, a marker measured largely by the same sandwich ELISA (Quidel A029 assay). Approximately 20-25% of the participants with home-based methods could not be analyzed due to differences in assay calibration/scale. Despite a good correlation over the past six years, every year there were outliers, especially in the upper range of the measurement scale with passing quota for both samples in the 65%-80% range.

Analysis for Bb was less informative, since participant numbers in the past six years varied from eight in 2017, to eleven, ten, eight, eight, seven, in the following years. During these years performance (passing quota) was 75%, 73%, 90%, 89%, 20% and 70%, respectively. Analysis for C3a and C3d was not feasible in the past years since participation remained constantly below eight laboratories.

### Performance of the participants for complement autoantibodies

EQA scheme EQA245 for determination of IgG anti-C1q autoantibodies was conducted twice a year, with a total cumulative participation of 69 laboratories. Approximately half of the participants used the same assay (provided by Orgentec), making it possible to analyze the performance separately from participants who used in-house assays or those from different providers (**Tables 1A, B**). In the Orgentec group, only 4/30 participants performed <80%, whereas in the in-house/INOVA group <80% performance occurred in 11/39 laboratories. It has to be mentioned that nearly half of the participants (14/30) in the Orgentec group, and 12/39 in the in-house/INOVA method group participated regularly, and performance among these frequent attendees was almost exclusively above 80% (bold facing in **Tables 1A**, **B**) in both groups.

**Table 1A:** Participation, passing quota and laboratory performance in the external quality assurance program EQA245 for anti-C1q IgG autoantibody (reagent: Orgentec).

Laboratory	2017 MARCH		2017 OCT		2018 MARCH		2018 OCT		2019 MARCH		2019 OCT		2020 MARCH		2020 OCT		2021 MARCH		2021 OCT		2022 MARCH	2022 OCT	Performance	Participation, total
1																							100%	1
2																							100%	4
3																							50%	2
4																							**100%**	7
5																							100%	2
6																							**91%**	11
7																							**89%**	9
8																							**89%**	9
9																							**91%**	11
10																							100%	4
11																							100%	4
12																							**80%**	10
13																							67%	9
14																							**100%**	7
15																							**100%**	8
16																							**100%**	7
17																							100%	4
18																							**80%**	10
19																							100%	1
20																							100%	3
21																							75%	4
22																							**89%**	9
23																							100%	1
24																							**91%**	11
25																							**91%**	10
26																							100%	5
27																							100%	5
28																							75%	4
29																							100%	2
30																							100%	1
**Passing quota**	100.0%		93.3%		88.9%		30.8%		93.3%		100.0%		100.0%		75.0%		93.8%		100.0%		100.0%	100.0%		
**Participation, total**	6		15		9		13		15		16		13		16		16		21		16	19		

Participation: Number of submitted results in the period of 2017-2022. Performance: Percentage of submitted results in the target range for both of the samples (dark blue). Any result out of target range (light red), lack of results (white). Passing quota: Performance of laboratories in the indicated surveys. Bold facing: laboratories with at least six participation and at least 80% performance.

**Table 1B:** Participation, passing quota and laboratory performance in the external quality assurance program EQA245 for anti-C1q IgG autoantibody (reagent: in-house or INOVA).

Laboratory	2017 MARCH		2017 OCT		2018 MARCH		2018 OCT		2019 MARCH		2019 OCT		2020 MARCH		2020 OCT		2021 MARCH		2021 OCT		2022 MARCH	2022 OCT	Performance	Participation, total
31																							100%	1
32																							80%	5
33																							0%	1
34																							100%	1
35																							100%	1
36																							**88%**	8
37																							**91%**	11
38																							100%	1
39																							100%	3
40																							**92%**	12
41																							100%	4
42																							100%	2
43																							**92%**	12
44																							0%	1
45																							100%	2
46																							100%	2
47																							50%	2
48																							100%	1
49																							73%	11
50																							100%	1
51																							50%	2
52																							**100%**	6
53																							**100%**	6
54																							**100%**	10
55																							**100%**	6
56																							100%	2
57																							50%	2
58																							50%	2
59																							0%	1
60																							75%	4
61																							**89%**	9
62																							0%	4
63																							100%	1
64																							**86%**	7
65																							**83%**	6
66																							50%	2
67																							100%	1
68																							100%	4
69																							50%	2
**Passing quota**	100.0%	83.3%		88.9%		30.8%		92.9%		90.0%		100.0%		69.2%		100.0%		76.9%		88.2%		86.7%		
**Participation, total**	8	12		9		13		14		10		18		13		17		13		17		15		

Participation: Number of submitted results in the period of 2017-2022. Performance: Percentage of submitted results in the target range for both of the samples (dark blue). Any result out of target range (light red), lack of results (white). Passing quota: Performance of laboratories in the indicated surveys. Bold facing: laboratories with at least six participation and at least 80% performance.

Interest for C3-nephritic factor (EQA248) increased over time with 28 laboratories participating at least once in the past seven years. Among these 28 laboratories 17 participated at least four times, but performance was above 80% for only 5/17 of the participants (bold facing in **Table 2**). For the remaining frequently participating laboratories performance was below 80%, and for laboratories with less than four participations proficiency was between 0% and 100%. No clear improvement or change in performance was noted in the past seven years. It has to be noted that participants used a large variety of methods for C3Nef determination. Due to the low number, even in the subgroup using the most frequently applied sheep red blood cell hemolysis based method it was impossible to compare the performance in subgroups discriminating for the applied method.

**Table 2:** Participation, passing quota and laboratory performance in the external quality assurance program EQA248 for C3-nefritic factor (C3Nef).

Laboratory	2016	2017	2018	2019	2020	2021	2022	Performance	Participation, total
1								25%	4
2								71%	7
3								0%	4
4								17%	6
5								57%	7
6								71%	7
7								**100%**	5
8								0%	2
9								100%	1
10								**86%**	7
11								**86%**	7
12								**86%**	7
13								0%	1
14								**100%**	4
15								43%	7
16								50%	4
17								0%	1
18								40%	5
19								100%	1
20								20%	5
21								0%	5
22								100%	3
23								0%	2
24								0%	1
25								50%	4
26								0%	2
27								33%	3
28								0%	1
**Passing quota**	81.8%	58.3%	43.8%	47.1%	31.3%	42.9%	70.0%		
**Participation, total**	11	12	16	17	16	21	20		

Participation: Number of submitted results in the period of 2016-2022. Performance: Percentage of submitted results in the target range for both of the samples (dark blue). Any result out of target range (light red), lack of results (white). Passing quota: Performance of laboratories in the indicated years. Bold facing: laboratories with at least four participations and at least 80% performance.

In contrast, interest for anti-FH didn’t change in the past years. 32 laboratories participated at least once, and performance was constantly above 80% (except for 2019) even four times above 90%. From fifteen frequently reporting laboratories, thirteen consistently performed well (bold facing in **Table 3**). The remaining seventeen participants with less than four participations showed highly variable results, with passing quota between 0% and 100%.

**Table 3:** Participation, passing quota and laboratory performance in the external quality assurance program EQA249 for anti-Factor H IgG autoantibody.

Laboratory	2016	2017	2018	2019	2020	2021	2022	Performance	Participation, total
1								**83%**	6
2								**86%**	7
3								**100%**	5
4								**100%**	7
5								100%	1
6								**100%**	4
7								0%	1
8								100%	1
9								**100%**	7
10								**100%**	7
11								**86%**	7
12								100%	1
13								**100%**	4
14								**86%**	7
15								100%	2
16								100%	1
17								0%	1
18								75%	4
19								**100%**	4
20								**100%**	7
21								**100%**	3
22								**100%**	5
23								67%	3
24								100%	3
25								100%	2
26								50%	4
27								100%	1
28								100%	1
29								0%	1
30								100%	1
31								100%	2
32								100%	1
**Passing quota**	92.3%	94.1%	94.4%	78.6%	88.2%	87.5%	93.8%		
**Participation, total**	13	17	18	14	17	16	16		

Participation: Number of submitted results in the period of 2016-2022. Performance: Percentage of submitted results in the target range for both of the samples (dark blue). Any result out of target range (light red), lack of results (white). Passing quota: Performance of laboratories in the indicated years. Bold facing: laboratories with at least four participations and at least 80% performance.

Finally, as summarized in **Table 4**, for IgG, IgA and IgM anti-C1-inhibitor autoantibodies (EQA250) interest was generally low (13 laboratories) with only six of thirteen taking part in more than three EQA rounds. None of them performed well for IgG, but six of six succeeded for IgA, and four of six for IgM.

**Table 4:** Participation, passing quota and laboratory performance in the external quality assurance program EQA250 for anti-C1-inhibitor autoantibodies.

Laboratory	2016	2017	2018	2019	2020	2021	2022	Performance	Participation, total
	IgG								
1								57%	7
2								20%	5
3								50%	4
4								0%	2
5								0%	1
6								57%	7
7								71%	7
8								100%	3
9								100%	1
10								17%	6
11								100%	2
12								100%	1
13								100%	2
**Passing quota**	42.9%	42.9%	85.7%	83.3%	42.9%	42.9%	42.9%		
**Participation, total**	7	7	7	6	7	7	7		
	IgA								
1								**100%**	7
2								**100%**	5
3								**100%**	4
4									0
5								100%	1
6								**100%**	7
7								**100%**	7
8								100%	3
9								100%	1
10								**100%**	5
11									0
12								100%	1
13								50%	2
**Passing quota**	100.0%	100.0%	100.0%	100.0%	100.0%	100.0%	83.3%		
**Participation, total**	7	6	6	6	7	5	6		
	**IgM**								
1								**86%**	7
2								**80%**	5
3								75%	4
4									0
5								100%	1
6								**100%**	7
7								**86%**	7
8								0%	1
9								100%	3
10								60%	5
11									0
12								100%	1
13								50%	2
**Passing quota**	85.7%	83.3%	100.0%	83.3%	85.7%	80.0%	50.0%		
**Participation, total**	7	6	6	6	7	5	6		

Participation: Number of submitted results in the period of 2016-2022. Performance: Percentage of submitted results in the target range for both of the samples (dark blue).Any result out of target range (light red), lack of results (white). Passing quota: Performance of laboratories in the indicated years. Bold facing: laboratories with at least four participations and at least 80% performance. Laboratory numbers in the three parts of the table indicate the same participants.

## Discussion

The need for a collaborative effort to monitor and improve the quality of complement testing was recognized in 2010. The successful introduction of an EQA was established only six years later as a complex and widely available program for which the results could be entered online. Such a program for analyzing the highly labile complement system presented a number of challenges, but by joining the expertise of the International Complement Society (ICS) with the knowledge and infrastructure of INSTAND e.V., a successful program could be initiated. This program not only gave an overview on the current state of complement diagnostic testing performance, but also provides the information necessary to improve complement testing procedures.

Our data demonstrates that the passing quota, across the assessments, is higher for normal samples than pathological samples. Looking first at functional analysis, the success rate for pathological samples in CP activity assessment demonstrates variations between the testing methods. Even for a given specific method, the passing quota varied between the years. For the first year the passing rate for both the normal and pathological samples analyzed by the hemolytic assay was below 90%, but in all subsequent years this method was most consistent, particularly for the pathological samples (**Figure 4**). Results of the ELISA initially had a lower passing quota for the pathological sample, but improved in recent years. This improvement may be attributed to the growing experience with this newer method. On the other side, the lower consistency of the non-specified method could be in part attributable to the lower number of laboratories reporting in this category. For the liposomal assay for CP activity in four rounds of testing (2020 and 2021), the passing quota for the pathogenic sample was ≤80%. These results are consistent with other publications suggesting that this method of measuring CP function is ideal for measuring low level activity (42, 43). However, it should be noted that in more recent rounds the passing quota for the pathological samples improved to greater than 90%. This is important because this method is more commonly used by standard hospital laboratories. Furthermore, a tighter clustering reflects less lab-to-lab variability in the reported results, an important consideration for comparability of testing results between laboratories (**Figure 5**).

AP activity is measured by fewer laboratories with less available testing methods. The passing quota for this analysis was overall lower than for the CP function especially for pathological samples. With the increasing recognition of its importance for disease development and drug monitoring the demand for this testing will certainly increase. Multiple AP specific therapeutic inhibitors are currently in Phase 3 clinical trials (44).

The overall passing quota for C1-INH function testing was higher and more consistent than those for CP or AP function measurements. Demand driven by need to follow therapeutic treatment may be part of the reason for the higher passing quota in testing C1-INHF. There are still method to method differences as is shown in **Figure 5**, where the reported results do group by method, but the spread of results is much tighter than for CP or AP function. Another contributing factor to the higher passing rate of C1-INHF may be the relative simplicity of testing the function of just one complement regulator, rather than a whole pathway.

The complexity of the complement CP and AP function tests is both their strength diagnostically, but also a potential cause of the observed variability. Their strength comes from the ability to evaluate eleven (CP) or nine (AP) different components in one test, respectively (45). For normal activity, all the components must be present and active. Any therapeutic inhibition along these pathways results in low or abnormal levels, also unraveling the complexities that arise from measuring the function of so many proteases at once. The relationship between protein concentration and activity of an individual component also relates to their drastically different concentrations in serum (from >1 g/L for C3 to 0.1 g/L for C1q, for example). Certain components are rate limiting and due to the stepwise nature of complement activation with several amplification steps the relationship between the component levels of the test serum and its activity is not strictly linear, but rather follows a Von Krogh equation (46). All current methods for measuring complement CP and AP function were developed for testing errors in inborn immunity and not for evaluating therapeutic inhibition of the complement system as now required. As more complement targeting drugs are approved, this may add pressure to the need on complement function testing.

In addition to the use of those functional assays, measurement of activation fragments is also growing in interest in response to the needs related to therapeutic interventions of the complement system. It is for this reason that the soluble membrane attack complex (sMAC, sC5b-9) has the highest participation rate of any of the activation markers. This complex has been proposed as a marker to better reflect that a patient responds to complement inhibition, or to assess if complement activation is causative for the clinical presentation (47–49). However, the utility of measuring sC5b-9 is not undisputed (50), probably also due an inconsistency of the measurements. In **Figure 6**, sC5b-9 data over six years of EQA assessment are shown. This analyte is only part of the October assessment and only reported by a minority of the participating laboratories. The passing quota of both the normal and pathologic samples reached 80% only once (2020) whereas most years it was only about 70% although testing was done in just one assay purchased from one manufacturer. The reason for this low passing quota is currently unclear, but may be explained by lab performance, or lot variations of the kit reagents, and low number of participants. It is unfortunate that the calibrator aimed to serve complement activation product assays (51) could not get more interest or acceptance in the past years, and the use is limited to a few laboratories. To this end, laboratories with divergent results are encouraged, as part of the EQA participation, to review their testing if their results do not receive a passing quota.

Of diagnostic importance is the measurement of autoantibodies which is hampered by the limited availability of sufficient quantities of appropriate samples for the complement related autoantibodies. As samples are taken from different patients in different years, variations in EQA results – probably also related to different methods applied- are not surprising. Specifically, the results for anti-C1q IgG autoantibodies in 2018 and 2020 demonstrated a notably lower level of agreement. This was true also for laboratories and methods that were otherwise highly consistent. The specific reasons for this discordance warrants further investigation, especially with reference to clinical presentation. In reviewing these results it is also important to keep in mind the low numbers of participating laboratories for some of these tests. When there are only a few laboratories reporting, individual results may have more impact on the overall passing quota.

The results presented for the complement autoantibodies exemplify an important practical shortage related to this field, i.e. how feasible it is for a small/new laboratory to introduce determination of for example anti-FH, anti-C1INH or C3Nef, as a new parameter. This difficulty is traced back to multiple factors, among which lack of international calibrators and control materials, and lack of commercial interest in these small diagnostic fields are the most important. The quality assessment group/committee already started to produce and share such control materials. One purpose of our article is to attract potential industrial partners and to improve the feasibility of the kit development.

A potential limitation of the current analysis is related to the fact that the test materials offered in this program are not exactly similar to that ones used in the daily routine work. This fact is related largely to logistic and financial aspects, however, during the initial elaboration of the program in the years between 2010 and 2016 efforts were done in the laboratories of the authors to identify the circumstances (in terms of recovery, stability and homogeneity) that are at the same time logistically feasible and technically sound. This is why lyophilization was introduced for three of the programs, and sample shipment at ambient temperature was accepted. However, these efforts make it not unnecessary to perform additional local control in the participating laboratories for preanalytical issues, while testing true routine samples.

Similar to other attempts undertaken to improve diagnostic immunology testing by the International Union of Immunological Societies (IUIS), the efforts of the ICS and INSTAND eV for complement testing is an important step towards improving its quality and standardization. With this view on the current state of testing our data are considered to empower the individual laboratories with knowledge for improvement of their performance, otherwise not available. At this more mature state of the EQA testing these data can facilitate international efforts to investigate how the current methods can be improved for better test results. Without such EQA data, it would be harder to identify the problems that need to be addressed, and any improvement would hardly be measurable.

## Data availability statement

The original contributions presented in the study are included in the article/**Supplementary Material**. Further inquiries can be directed to the corresponding author.

## Ethics statement

The studies involving humans were approved by Hungarian Ethical Review Agency (ETT-TUKEB). The studies were conducted in accordance with the local legislation and institutional requirements. The participants provided their written informed consent to participate in this study.

## Author contributions

MK: Conceptualization, Methodology, Supervision, Validation, Writing – original draft, Writing – review & editing, Investigation. AF-A: Conceptualization, Investigation, Methodology, Supervision, Validation, Writing – original draft, Writing – review & editing. EB: Methodology, Validation, Writing – original draft, Writing – review & editing, Resources. SG: Data curation, Formal analysis, Methodology, Project administration, Validation, Writing – original draft, Writing – review & editing. NW: Conceptualization, Data curation, Investigation, Methodology, Project administration, Validation, Writing – original draft, Writing – review & editing. ZP: Methodology, Validation, Writing – original draft, Writing – review & editing, Conceptualization, Formal analysis, Project administration, Supervision, Visualization.
